# Intratumoral genetic and immune microenvironmental heterogeneity in T4N0M0 (diameter ≥ 7 cm) non‐small cell lung cancers

**DOI:** 10.1111/1759-7714.14393

**Published:** 2022-04-08

**Authors:** Jia−Tao Zhang, Song Dong, Li−Yan Ji, Jia−Ying Zhou, Zhi− Hong Chen, Jian Su, Qing−Ge Zhu, Meng−Min Wang, E−E. Ke, Hao Sun, Xue−Tao Li, Jin−Ji Yang, Qing Zhou, Xu− Chao Zhang, Xuan Gao, Xue−Ning Yang, Xuefeng Xia, Xin Yi, Wen−Zhao Zhong, Yi−Long Wu

**Affiliations:** ^1^ The Second School of Clinical Medicine Southern Medical University Guangzhou China; ^2^ Guangdong Lung Cancer Institute, Guangdong Provincial People's Hospital Guangdong Academy of Medical Sciences Guangzhou China; ^3^ Geneplus‐Beijing Institute Beijing China

**Keywords:** chromosomal instability, clonal structure, immune microenvironment, intratumoral heterogeneity, metastasis

## Abstract

**Background:**

Starting with low metastatic capability, T4N0M0 (diameter ≥ 7 cm) non‐small cell lung cancers (NSCLCs) constitute a unique tumor subset, as with a large tumor size but no regional or distant metastases. We systematically investigated intratumoral heterogeneity, clonal structure, chromosomal instability (CIN), and immune microenvironment in T4N0M0 (≥7 cm) NSCLCs.

**Methods:**

Whole‐exome sequencing, RNA sequencing, and multiplex immunohistochemistry (mIHC) staining were conducted to analyze 24 spatially segregated tumor samples from eight patients who were pathologically diagnosed with T4N0M0 (diameter ≥ 7 cm) NSCLCs. The adjacent normal tissues and peripheral blood served as controls.

**Results:**

In total, 35.2% of mutations and 91.1% of somatic copy number alterations were classified as subclonal events, which exhibited widespread genetic intratumoral heterogeneity. In contrast, a low degree of CIN was observed. None of the patients had genome doubling. The burden of loss of heterozygosity, aneuploidy, and the genome instability index of these tumors were significantly lower than those in the TRACERx cohort. Expression profiles revealed significantly upregulated expression of cell division‐related signals and the G2/M checkpoint pathway. In addition, a similar expression pattern of the immune microenvironment was observed in different regions of the tumor, which was confirmed by mIHC profiles.

**Conclusions:**

Our study indicates the presence of intratumoral genetic heterogeneity and immune microenvironmental heterogeneity features in T4N0M0 NSCLCs, and the low degree of CIN may be related to the low metastatic capability.

## Graphical abstract

A subset of non‐small cell lung cancer that exceed 7 cm in diameter but lack regional lymph node or distant organ metastases (stage T4N0M0) represent a distinct class of tumors with low metastatic capability. Intratumoral genetic and immune microenvironment heterogeneity were systematically analyzed of 24 spatially segregated tumor samples. This study highlights the low degree of CIN may be related to its low metastatic capability.

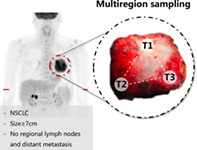

## INTRODUCTION

Unlimited proliferation is a principal characteristic of cancer cells. Accordingly, tumor progression and metastasis remain the major causes of cancer‐related mortality.^1^ However, over the course of tumor evolution, a proportion of non‐small cell lung cancers (NSCLCs) may exhibit a low metastatic capacity at a particular stage. A well‐recognized example is localized pleural seeding observed unexpectedly during surgery (s‐pM1a). Several studies have reported that s‐pM1a NSCLCs inherit a profound prognosis and lower metastatic ability than other stage IV NSCLCs.^2^, ^3^, ^4^, ^5^, ^6^ Nevertheless, the underlying genomic features of this biological behavior have not been elucidated.

In recent years, multiomics analysis of intratumoral heterogeneity (ITH) has revolutionized our understanding of the molecular and genetic bases of cancer development and evolution. In particular, the Tracking Non–Small‐Cell Lung Cancer Evolution through Therapy (TRACERx) project has provided critical insight into the intrinsic driving force of chromosome instability (CIN) in ITH, which contributes to an increased risk of recurrence or death.^7^ Several studies have further expounded on the relationship between tumor invasiveness and CIN, including whole‐genome or segmental duplication, loss of heterozygosity, and somatic copy number (CN) variation.^8^, ^9^, ^10^, ^11^, ^12^ Additionally, recent studies have highlighted the critical role of the imbalance between tumor and host immunity in tumor progression, a process termed as immune escape.^13^ Furthermore, novel routes to immune evasion are being discovered, including T cell exhaustion,^14^ depletion of expressed neoantigens,^15^ and loss of human leucocyte antigen (HLA).^16^ Therefore, analyses of ITH, CIN and immune microenvironment of NSCLCs with low metastatic capability will facilitate our knowledge of tumor growth and invasiveness.

In clinical practice, a subset of NSCLCs that exceed 7 cm in diameter and lack regional lymph node or distant organ metastases (stage T4N0M0) is occasionally observed. Patients with T4N0M0 NSCLCs present exhibited a relatively favorable prognosis after radical resection, with a 47% 5‐year survival rate.^17^, ^18^ Hence, T4N0M0 (≥7 cm) NSCLCs could constitute a distinct class of tumors with low metastatic capability. Nevertheless, the genomic characteristics of this class of tumors have not been defined. In this study, we harnessed a multifaceted approach comprising whole‐exome and transcriptome sequencing in addition to multiplex immunohistochemistry (mIHC) by multiregion sampling to systematically investigate the ITH, clonal structure, CIN, and immune microenvironment of T4N0M0 (≥7 cm) NSCLCs.

## METHODS

### Patients and tumor samples

We prospectively enrolled patients who were diagnosed with stage T4N0M0 (≥7 cm) NSCLCs and underwent surgical resection. Preoperative positron emission tomography‐computed tomography staging, and postoperative pathological diagnosis confirmed that none of the patients had regional lymph node or distant metastasis. The clinicopathological features of the enrolled patients are summarized in Table S1.

**FIGURE 1:**
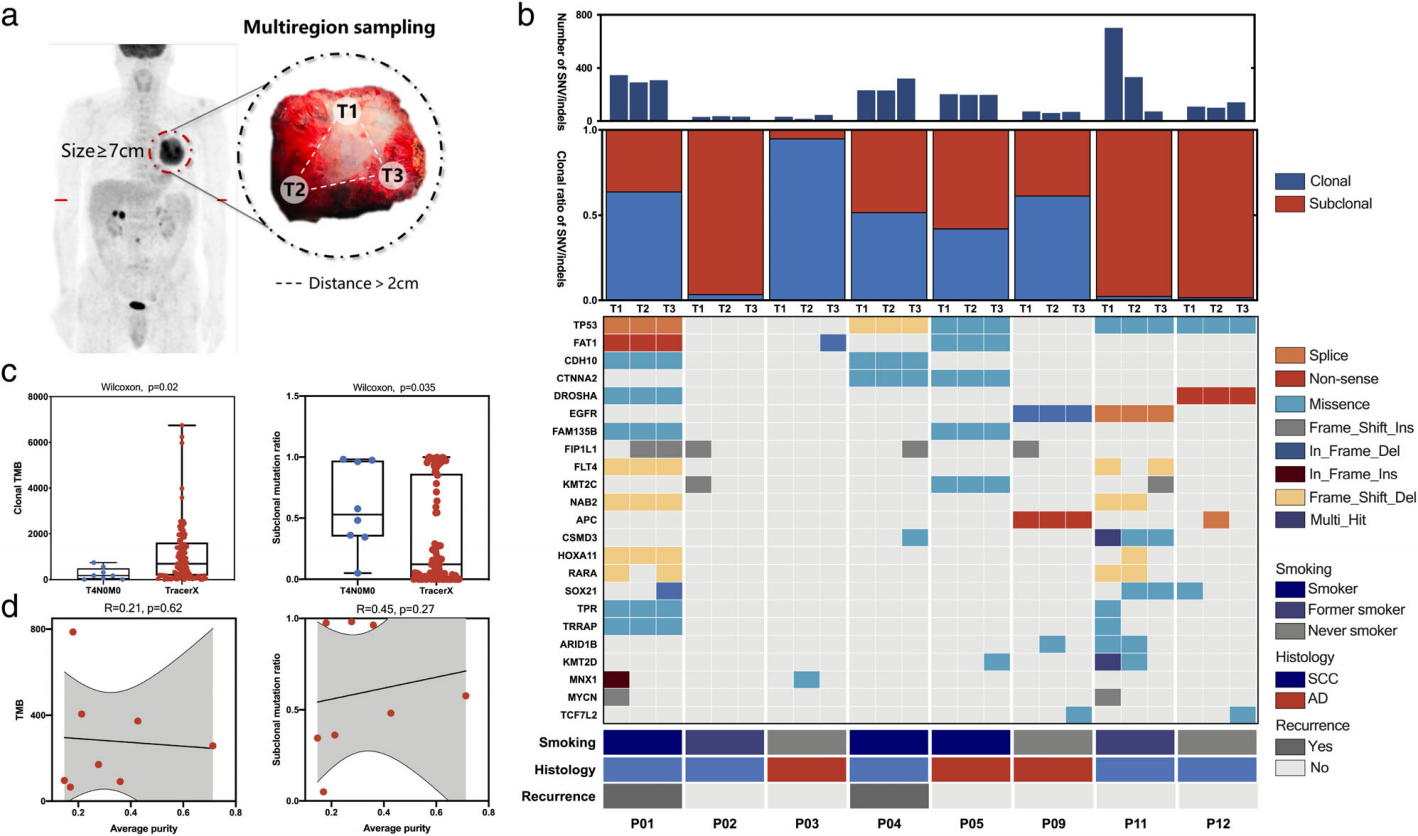
Genomic heterogeneity and clonal structure. (a) Schematic diagram of multiregion sampling of tumors over 7 cm in diameter without lymph node or distant metastasis. (b) Clonal structure of nonsynonymous mutations and a heatmap diagram of driver mutations and clinical features in each tumor region. (c) Clonal mutation burden and subclonal mutation ratio of T4N0M0 NSCLC versus TRACERx tumors. (d) Correlation analysis of tumor mutation burden or subclonal mutation ratio with average tumor purity. NSCLC, non‐small cell lung cancer

In total, 24 fresh tumor tissues and matched adjacent normal tissues were collected from all eight patients during surgery. Peripheral blood was used as the control (Figure S1). To assess ITH, three different regions separated by at least 2 cm (Figure 1a) were sampled per tumor. Large patches of necrotic regions were avoided; tumor samples were washed in phosphate‐buffered saline to exclude residual necrotic material. The TRACERx study was used as an external cohort.^19^ Whole‐exome sequencing was performed on DNA samples from the same tissue, as previously described by Jamal‐Hanjani et al.^7^

This study was approved by the Institutional Review Board of the Guangdong Provincial People's Hospital (Approval no. GDREC2019523H). Written informed consent was obtained from all patients.

### **Multiregion** **whole‐exome sequencing**

DNA and RNA were extracted using the Qiagen All Prep DNA/RNA FFPE kit or separately with QIAamp DNA FFPE Tissue Kit and RNeasy FFPE kit, respectively. The DNA extracted from multiple regions and collected from each tumor was prepared to generate a library with dual‐unique molecular identifiers for the MGISEQ‐2000 platform with paired‐end 100 bp. Raw sequencing data were removed from low‐quality reads and low‐quality bases using fastp,^20^ mapped to the human genome (hg19) using BWA‐MEM (bwa‐4.0.8.1) and sorted. Duplications were removed separately using samtools (v1.3.1) (http://samtools.sourceforge.net) and Picard (2.6.0) (https://broadinstitute.github.io/picard/). Neoantigens were predicted with netMHCpan‐3.0.^21^

### Somatic mutation calling

TNscope (https://www.sentieon.com/) was employed to detect somatic mutation variants, insertions, and deletions (indels) with default parameters based on paired alignment BAM files. Mutations were filtered out if they met the following criteria: variant allele fraction (VAF) of <0.03 and rare variant fraction of ≧0.01 in databases, including ExAC, ESP6500, dbSNP, and 1000G. Mutations identified in one or two regions were rescued in other regions with the following filters: support by both strands, vaf ≧0.01, total mutant reads ≧5, and supporting ≧30× depth at loci in tumors; and normal reads ≥10 with mutant reads ≤5 and vaf ≤0.01 in normal tissues. Variant classifications, including splice‐site, nonsense mutations, missense mutations, frameshift, and in‐frame indels, were reserved. Cross‐contamination among patients was assessed using hierarchical clustering of Spearman correlations of germline single‐nucleotide variants (SNVs) between samples.^22^

### Chromosomal aberration detection

Somatic copy number alterations (SCNAs) were estimated using FACETS.^23^ Ploidy, purity, and loss of heterozygosity (LOH) were detected using the R package ABSOLUTE.^24^ Significant SCNAs and arm‐level SCNAs were detected using GISTIC2.^25^ Whole‐genome doubling (WGD) was estimated using the algorithm developed by McGranahan.^9^ In particular, the observed CN gain or loss was compared to the simulated CN gain or loss 10,000 times. The sample was considered to experience WGD if *p* < 0.001 at diploid or triploid, *p* < 0.05 at tetraploid, *p* < 0.5 at pentaploid, or *p* ≦ 1 at hexaploid.^9^

CN gain was defined as CN/ploidy ≥2.5/2, whereas CN loss was defined as CN/ploidy ≤1.5/2.^7^ The genome instability index was defined as the ploidy‐corrected ratio of regions with CN gain or loss to the whole‐genome length.^26^

### Clonal structure analysis

All somatic nonsilent mutations and SCNAs were used to construct clone structures using Pyclone‐VI.^27^ Clonal mutations were defined as those in the cluster with the highest cellular cancer frequency. Other mutations were set as subclonal mutations. For clonal SCNAs, SCNA gain or loss occurring in all three regions within tumors was defined as a clonal somatic copy number variation. SCNA gain or loss observed in only one or two regions within tumors was defined as subclonal SCNAs.

### Gene expression data analysis

mRNA libraries were prepared using the NEBNext Ultra RNA Library Prep Kit for Illumina according to the manufacturer's protocol. RNA‐seq libraries were paired‐end sequenced on an MGISEQ‐2000 sequencer. Sequencing reads containing adaptor sequences and low‐quality reads were filtered, and 24.2–81.4 Mb clean reads were mapped to hg19 using STAR v2.7.5. Differential analysis was performed using DESeq2 and EdgeR (|fold change| ≥ 2 and *p* < 0.05). Pathway enrichment was performed using clusterProfiler^28^ and gene set enrichment analysis (GSEA).^29^ Quantitative measurements of immune cell infiltration were generated using single‐sample GSEA (ssGSEA),^30^, ^31^ which has been applied in several studies to infer the relative level of immune cell infiltration based on RNA profiling data.^32^, ^33^

Immune cell scores and stromal scores were determined using the ESTIMATE R package.^34^ Immune cell infiltration was estimated using the TIMER2 platform (http://timer.cistrome.org/) using multiple algorithms, including TIMER, CIBORSORT, CIBORSORT‐ABS, MCPcounter, and QUANTISEQ.^35^ Immune cells were assessed with markers reported by Danaher^36^ and CD8 cell markers^15^ using ssGSEA. Immune‐related gene sets were determined based on relevant published references, such as those for antigen presentation,^15^ chemokines,^37^ and T cell inflammatory genes.^38^

### Multiplex immunohistochemistry (mIHC) staining

The microenvironment of all tumor samples and adjacent normal tissues was comprehensively assessed using the Akoya Opal seven‐color fluorescent platform. All sections were stained using the Opal Polaris 7 Color Automation IHC Detection Kit (Akoya Biosciences) for the simultaneous detection and quantification of Pan‐CK, CD8, FoxP3, PD‐1, Granz‐B, Ki‐67, and DAPI (Table S2).

### Statistical analysis

All statistical analyses were conducted using R4.0.2 (https://www.r-project.org/). Quantitative data were analyzed using the Mann–Whitney U test for comparisons between groups. Spearman correlation analysis was performed to assess the associations between the samples. Statistical significance was set at *p* < 0.05.

## RESULTS

### High levels of intratumoral heterogeneity

Based on the low metastatic capacity of these tumors, we hypothesized that a higher homogeneity would be observed. Curiously, an extremely high level of ITH was observed.

In total, 24 samples were sequenced, with three regions from each patient (mean depth, 378×). Overall, 4175 SNVs and indels were identified, affecting the exons of 1787 genes (Figure 1b). We identified 181 driver events (median, 19.5; range, 5–49) based on previously reported criteria (Figure 1b).^39^ Of SNV/indels, 35.2% were identified as subclonal mutations. The average subclonal ratio of SNVs/indels exceeded 80% in three of them, P02 (85.1%), P11 (90.4%), and P12 (91.7%), in contrast with the data reported in previous studies of subclonal mutation ratios in lung cancer (~30%) (Figure 1b).^7^ Further analysis revealed that the subclonal mutation ratio of these T4N0M0 NSCLCs was significantly higher than that in the TRACERx cohort (Figure 1c).

**FIGURE 2:**
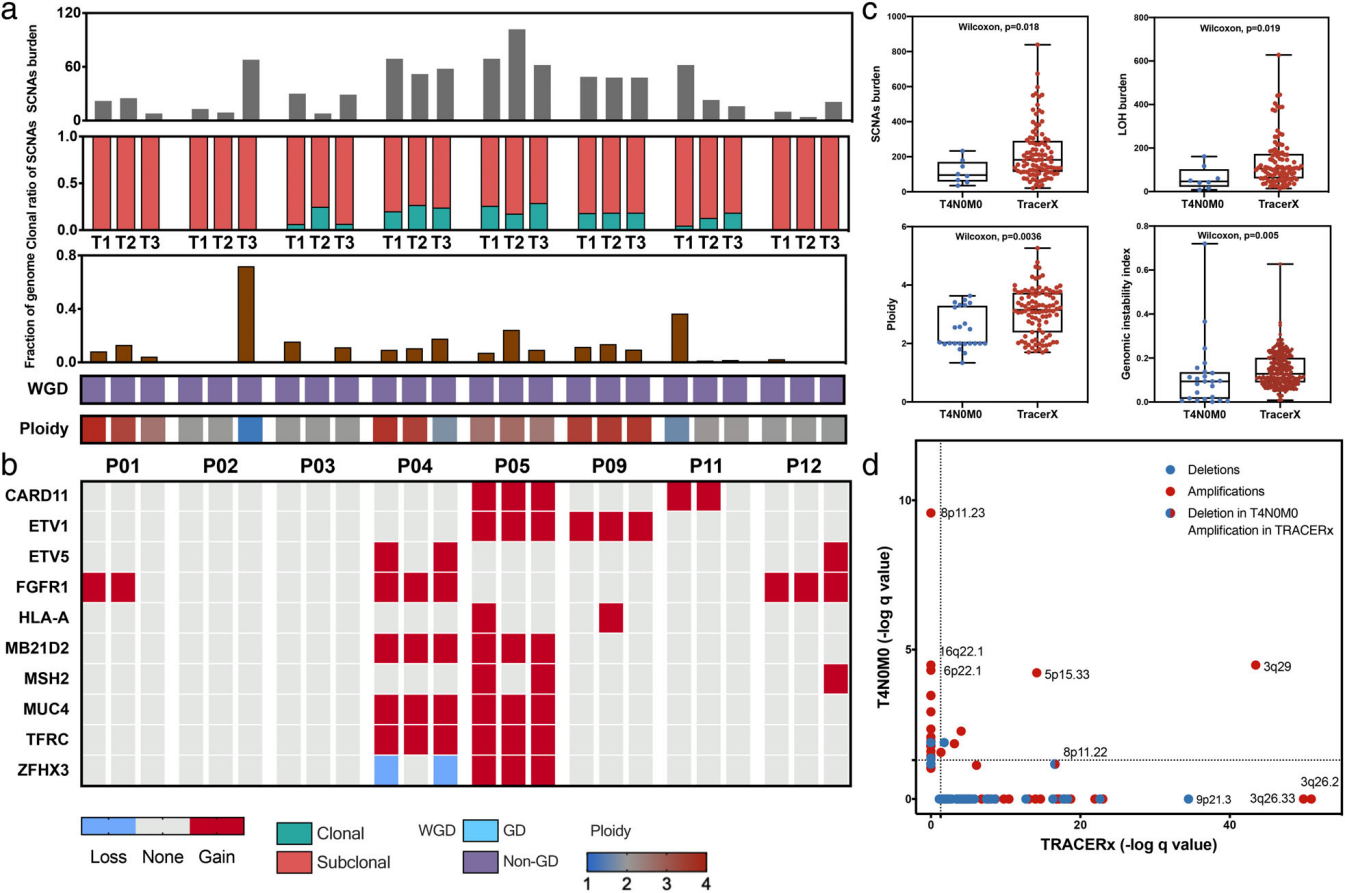
Intratumoral heterogeneity of somatic copy number alterations (SCNAs) and chromosomal instability. (a) Number and clonal to subclonal ratio of SCNAs in each tumor region. The ploidy‐corrected fraction of the genome altered by SCNAs, which is defined as the genomic instability index (GII), is presented in the figure. Whole‐genome doubling status and genome ploidy are also presented. (b) Heatmap diagram of SCNAs occurring in at least two tumor regions. (c) SCNA burden, loss of heterozygosity (LOH), ploidy, and GII for T4N0M0 NSCLCs versus TRACERx tumors. (d) Amplification (red) and deletion (blue) *q* values from GISTIC2.0 for SCNA peaks of significant copy number gain and loss plotted for T4N0M0 NSCLCs versus TRACERx tumors. NSCLC, non‐small cell lung cancer

SCNAs of all tumor sections were successfully profiled. In total, 905 SCNA events were identified (median: 95.5, range: 35–233, Figure 2a,b), including 426 gains and 479 losses present in at least one tumor region. A median of 91.1% was identified as subclonal SCNAs (Figure 2a), which was higher than the 28%–48% of NSCLCs previously reported.^7^, ^10^ Moreover, none of the SCNAs were shared among the three regions as trunk events for P01, P02, and P12.

In consideration of misidentified and cross‐contaminated tumor samples, we mapped the genetic distances between all samples to verify their identity. All patient‐specific genomic DNA samples clustered together, as expected (Figure S2). Another concern was that the extremely high ITH and variable mutation burden may have been a misestimate due to the tumor purity in different tumor regions. However, no significant correlation between tumor purity and tumor mutational burden or subclonal mutation ratio was observed (Figure 1d; Figure S3).

### Low degree of chromosomal instability

Since CIN is considered a major driver of ITH and shapes tumor evolution,^8^, ^40^ we hypothesized that a high degree of CIN would be observed. Using the genome instability index (GII, defined as the fraction of the genome altered by SCNAs, corrected by ploidy), we identified that the majority of the tumors exhibited low instability (median of 9.5% per tumor, Figure 2a,b), in contrast to previous findings of lung adenocarcinoma (~48%).^26^ Given that WGD events are associated with the propagation of CIN,^10^ we next examined the WGD status of these indolent tumors. As expected, none of the tumors exhibited WGD (Figure 2a), in contrast with the information in a previous report (59% for lung adenocarcinoma and 55% for lung squamous cell carcinoma).^41^ We further compared these T4N0M0 NSCLCs with data from the TRACERx cohort with regard to different aspects of genomic instability, including SCNA burden, LOH, ploidy, and GII. We observed significantly lower values in different dimensions in these tumors than those in the TRACERx cohort (*p* < 0.05, Figure 2c). Furthermore, we employed GISTIC 2.0 to identify statistically significant recurring SCNAs between our cohort and patients with lymph node metastasis in the TRACERx cohort (Figure 2d). We identified 37 significant regions in the indolent T4N0M0 tumors, 26 amplifications, and 11 deletions, of which 20 amplifications and nine deletions were not identified in the TRACERx cohort (e.g., 8p11.23), suggesting relative specificity for these T4N0M0 tumors (Figure S4A). Similar trends of amplification in 8p11.23 were also shown when adenocarcinomas and squamous cell carcinomas are separate (Figures S4B,C). Of note, converse CN changes between the two groups were observed in 8p11.22, which had downregulated expression in T4N0M0 tumors but upregulated expression in the TRACERx cohort. We further compared gene expression levels between tumors and adjacent normal tissues encompassing 8p11.22 and 8p11.23 (Figure S5). The results indicated that the expression patterns of *ADAM32*, *TACC1*, and *C8orf4* were consistent with chromosomal region deletions (8p11.22).

### Upregulated cell division‐related signals and enriched G2/M checkpoint

**FIGURE 3:**
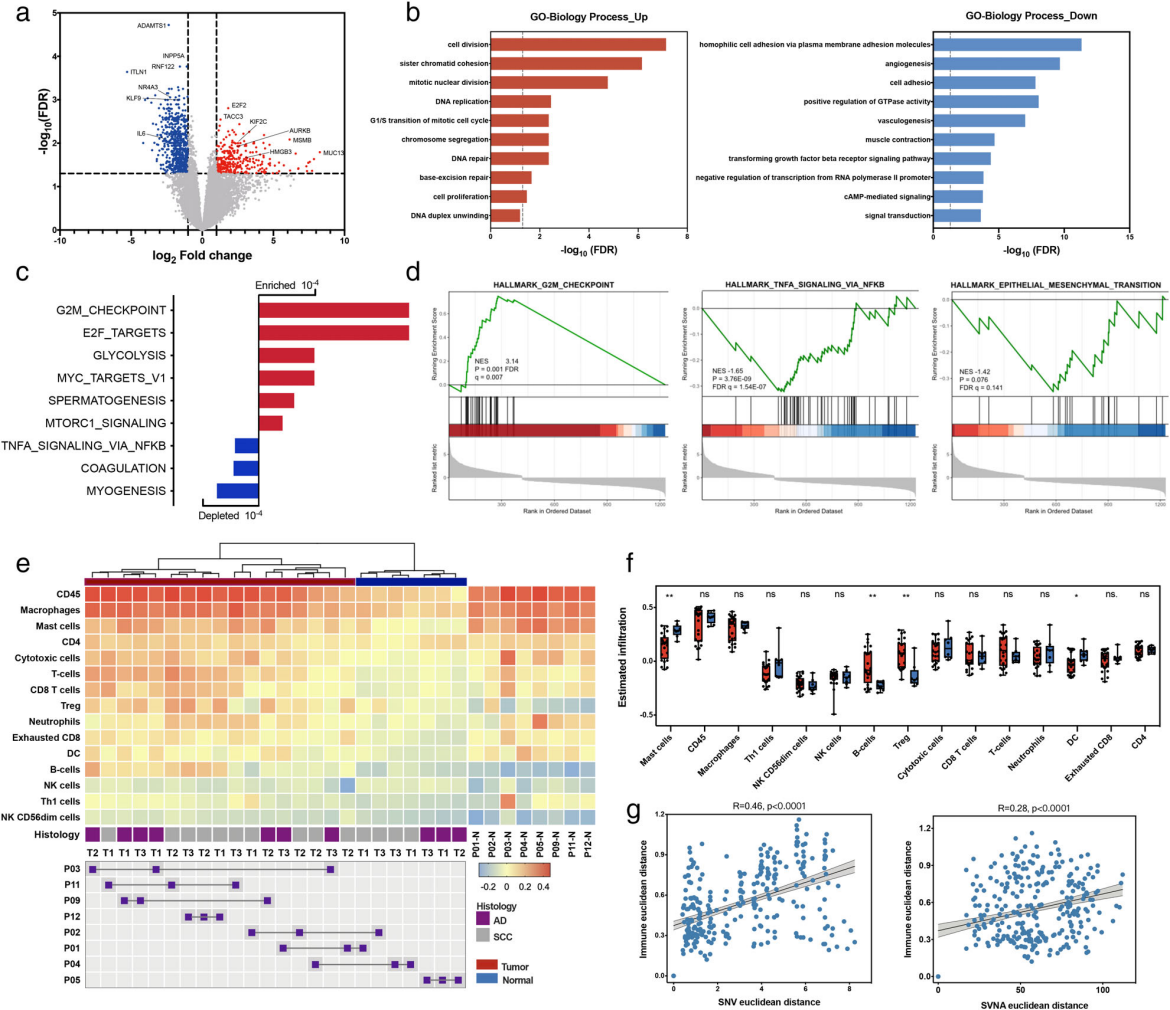
Expression profiles. (a) Volcano plot of differentially expressed genes between tumors and adjacent normal tissues. (b) Gene ontology (GO) analysis of genes with upregulated (red) and downregulated (blue) expression involved in biological processes. (c, d) Summary of gene set enrichment analysis (GSEA) and plots of representative data. (e) Clustering heatmap of the estimated immune infiltrates. Each row represents the population of immune cells. The intratumor heterogeneity of the estimated immune infiltrates and different regions for the same patient are connected with lines. (f) Abundance of different immune cell types between tumors (red) and adjacent normal tissues (blue). (g) Comparison of pairwise genomic and immune distances between every two tumor regions from the same patient

In total, 1230 differentially expressed genes (DEGs) were identified between tumor regions and adjacent tissues, consisting of 416 upregulated genes and 814 downregulated genes (Figure 3a). The DEGs were annotated with gene ontology terms based on biological processes (Figure 3b). Particularly, genes associated with cell division‐related signals, including signals for cell division, sister chromatid cohesion, mitotic nuclear division, DNA replication, and chromosome segregation, were highly expressed in tumor samples (Figure 3b). In addition, genes associated with DNA repair and base‐excision repair had upregulated expression (Figure 3b). GSEA revealed enrichment of the G2/M checkpoint and E2F‐target signatures (Figure 3c). Checkpoints occur at entry into mitosis (the G2/M checkpoint), which provides time for DNA repair through arrest or delay of cell cycle progression.^42^, ^43^ Hence, we speculated that vigorous mitotic activity may be a major factor contributing to the large tumor volume. In this regard, the DNA repair ability of these tumors remained at a relatively high level via the G2/M checkpoint pathway. Enrichment in the epithelial‐to‐mesenchymal transition pathway was not observed in GSEA, which is consistent with the low metastatic capability of these indolent tumors (Figure 3d).

### Heterogeneity of tumor immune microenvironment (TIME)

**FIGURE 4:**
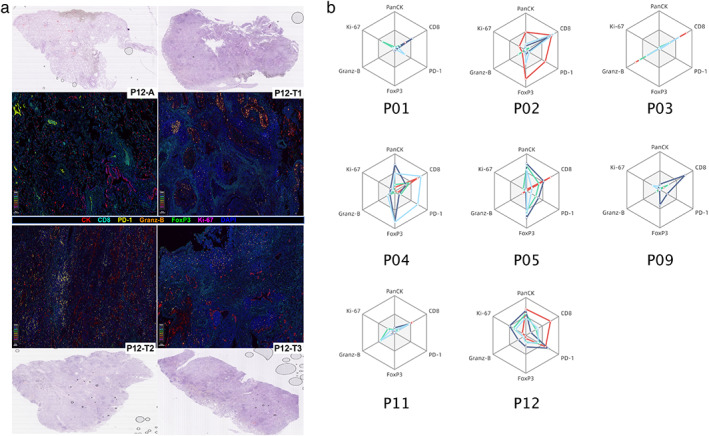
Tumor immune microenvironment profiling with fluorescent multiplex immunohistochemistry (mIHC). (a) Representative mIHC images of P12, including adjacent normal tissue (P12‐A) and three separate tumor regions (P12‐T1, T2, and T3). (b) Quantitative radar plots of the positivity of six markers in each patient, including Pan‐CK, CD8, PD‐1, FoxP3, Granz‐B, and Ki‐67. Adjacent normal tissue (red), T1 (green), T2 (dark blue), and T3 (light blue). Data were transformed into log(1 + positivity), and the axes represent 0 to 2 from the inner to the outer ring

Next, we explored the TIME using expression data and mIHC profiles. Using ssGSEA, we estimated the RNA‐seq‐derived infiltrating immune cell composition of 32 tissue samples (Figure 3e). Two distinct immune clusters corresponding to higher and lower levels of immune infiltration, respectively, were identified in the clustering analysis. Additionally, most of the tumor samples had a similar level of immune infiltration (e.g., P12 and P05). The low degree of TIME heterogeneity was further confirmed by the mIHC profiles (Figure 4a). Compared with adjacent normal tissue (red), different tumor regions from same patient showed similar expression pattern of immune markers (Figure 4b).

We further compared the abundance of different immune cell types between tumors and adjacent normal tissues. A significantly higher abundance of infiltrating Treg and B cells and a lower abundance of mast cells and dendritic cells were observed in the tumor area (*p* < 0.01, Figure 3f). Further comparison using other methods (TIMER, CIBORSORT, MCPcounter, and others) confirmed the higher abundance of B cells infiltrating the tumor tissue, predominantly comprising naïve and memory B cell phenotypes (Figure S6).

We also observed a significant correlation between the two pairwise distance measures (Figure 3g, *p* < 0.0001), supporting an interplay between immune and cancer genomic landscapes and highlighting the distinct immune microenvironments in tumor regions distant in genomic space, in agreement with the results of the TRACERx study.^15^

## DISCUSSION

Growing evidence suggests that a subset of lung tumors, including oligometastatic tumors, s‐pM1a NSCLCs, and pulmonary nodules with ground glass features may exhibit low metastatic behavior in certain contexts. Owing to the diversity in clinical contexts, establishing a standardized definition for indolent lung cancer has been challenging. In this study, we enrolled eight patients with primary tumors exceeding 7 cm in diameter but without regional lymph nodes or distant organ metastasis (T4N0M0). Overall, our multiomics integration analysis demonstrated that landscape of genomic and immune microenvironment heterogeneity of this subset of tumors. Moreover, we highlighted the features of high ITH, low CIN and similar pattern of immune infiltration.

Studies such as the PCAWG project have contributed to the growing evidence supporting the role of CIN during tumor invasion and metastasis. A large pan‐cancer analysis on CIN by Watkins et al. revealed that most recurrent arm‐level SCNA events were enriched in metastatic samples, which contributed to the metastatic potential of the tumor.^10^ For example, two loss regions (17p13.3–q11.2 and 19p13.3) were significantly enriched in lung adenocarcinoma metastases, which may have been associated with their metastatic potential.^10^ This evidence was supported by another largescale whole‐genome study which profiled data from 2520 metastatic tumors. Based on a comparison with primary tumors in the PCAWG project, the authors concluded that there were no fundamental genomic differences between metastatic tumors and primary tumors in terms of the mutational landscape or genes driving advanced tumorigenesis. However, several CIN‐related genomic features were enriched in metastatic tumors. WGD affected 56% of all metastatic cancers, and an average of 23% of the autosomal DNA exhibited LOH. Furthermore, up to 80% of tumor‐suppressor genes were inactivated biallelically. These findings support the relationship between CIN and the metastatic ability of tumors. Similar findings were noted in our study: the degree of CIN in T4N0M0 tumors was significantly lower than that in the TRACERx data, including WGD, ploidy, LOH, and GII.

Notably, an extremely high degree of ITH was observed in these T4N0M0 NSCLCs, with 35.2% of SNV/indels and 91.1% of SCNAs were identified as subclonal events. This could be partly due to the low proportion of tumor cells in the tumor bed of these large tumors as calculated by ABSOLUTE and ESTIMATE. Furthermore, the mIHC staining confirmed the relatively low positive rate of Pan‐CK (Figure S7). This bias may also interfere with small sample size or the clone structure algorithm that we used. Moreover, CIN is presently thought of as a major driver of ITH,^7^ but it exhibits the opposite trend between CIN and ITH in these T4N0M0 tumors. The reason for this paradox is unclear. Nevertheless, we can speculate that the metastatic or invasive capability of these T4N0M0 tumors is not related to their high degree of ITH but rather the low CIN.

Intratumoral heterogeneity of TIME in lung cancer has been explored in previous studies. The ITH of programmed death‐ligand 1 (PD‐L1) expression level has been observed in previous studies.^44^, ^45^, ^46^ Jia et al. also used multiomics analysis for different tumor regions in 15 NSCLC patients and showed the heterogeneous features of immune niches within NSCLC tumors, so called “immunologically hot area” and “immunologically cold area.”^33^ In our study, we observed a similar pattern of immune infiltration of these T4N0M0 tumors. However, the effect of TIME heterogeneity on the efficacy of immune checkpoint inhibitor requires further exploration.

There were two patients relapsed until December 2021. We further compared the ITH, CIN and TIME features between patients who relapsed and who did not. Despite limitation in sample size, no significant differences were seen on ITH and CIN features, including subclonal ratio of SNVs/indels, SCNAs burden, LOH, ploidy and GII. However, the CD4+ and CD8+ T cells appear to be more prevalent in the tumor area from patients who did not relapse (Figures S8A–C). Moreover, there were not significantly different when we compared the adenocarcinoma and squamous cell carcinoma (Figure S8D).

Our study had two major limitations. First, the sample size was small; as neoadjuvant therapy is one of the optimal options for stage IIIA NSCLCs, sample collection for untreated T4N0M0 NSCLCs is made more difficult. Second, a valid control group was missing in our study. Although a comparison with the TRACERx cohort was performed in our analysis, the potential presence of confounding factors cannot be ruled out.

In conclusion, we investigated the ITH, CIN and TIME of a specific cohort of NSCLCs in this study. The low degree of CIN may be related to the low metastatic capability of T4N0M0 lung tumors. Further studies are required to expand upon and verify our results.

## CONFLICT OF INTEREST

Qing Zhou declares speaker fees from AstraZeneca and Roche. Wen‐Zhao Zhong declares speaker fees from AstraZeneca and Roche. Yi‐Long Wu declares speaker fees from AstraZeneca, Eli Lilly, Pfizer, Roche, and Sanofi. None of the other authors have any conflicts of interest to declare.

## Supporting information


**Figure S1.** Sequencing and analytical flowchart of the study. mIHC, multiplex immunohistochemistry; WGD, whole‐genome doubling; LOH, loss of heterozygosity; SCNA, somatic copy number alterations; GII, genomic instability index; TIME, tumor immune microenvironment.
**Figure S2**. Cluster map of genetic distances between all samples.
**Figure S3**. Correlation between somatic copy number alterations (SCNAs) and average tumor purity.
**Figure S4**. (A) GISTIC amplification (top, red) and deletion (bottom, cyan) plots of the T4N0M0 tumor cohort (dark) and TRACERx cohort (light). (B) Amplification (red) and deletion (blue) *q* values from GISTIC2.0 for SCNA peaks of significant copy number gain and loss plotted for T4N0M0 adenocarcinomas versus TRACERx adenocarcinomas (LUAD, *n* = 61). (C) Amplification (red) and deletion (blue) *q* values from GISTIC2.0 for SCNA peaks of significant copy number gain and loss plotted for T4N0M0 squamous cell carcinomas (LUSC) versus TRACERx LUSCs.
**Figure S5**. Gene expression levels of tumors (red) and adjacent normal tissues (blue) encompassing 8p11.22 and 8p11.23.
**Figure S6**. Estimated proportion of infiltrating immune cells in tumors (red) and adjacent normal tissues (blue) obtained with MCPcounter, TIMER, QUANTISEQ, CIBORSORT, and CIBORSORT‐ABS.
**Figure S7**. (A) Positive results of different mIHC markers between tumor regions and non‐tumor regions based on Pan‐CK signal. (B) Correlation analysis of tumor purity or ESTIMATE ratio with a Pan‐CK positive result.
**Figure S8**. Subgroup analysis. (A) Subclonal mutation and somatic copy number alterations (SCNAs) ratio. (B) Genomic instability index, loss of heterozygosity (LOH), SCNAs burden and ploidy. (C) Abundance of different immune cell types estimated by ssGSEA methods, Relapse (*n* = 2) versus nonrelapse (*n* = 6). (D) Abundance of different immune cell types estimated by ssGSEA methods, lung adenocarcinoma (LUAD, *n* = 3) versus squamous cell carcinoma (LUSC, *n* = 5). The asterisk indicates relapsed patients and the hashtag indicates LUAD patients.

**Table S1.** Clinicopathological features of all eight enrolled patients.
**Table S2**. Multiplex immunohistochemistry markers.
