# Supplementary material for: Intratumoral genetic and immune microenvironmental heterogeneity in T4N0M0 (diameter ≥ 7 cm) non‐small cell lung cancers
Source: Thorac Cancer. 2022 Apr 8;13(9):1333–41. doi: 10.1111/1759-7714.14393 (PMC9058296; doi:10.1111/1759-7714.14393)

Supplementary Figure 1

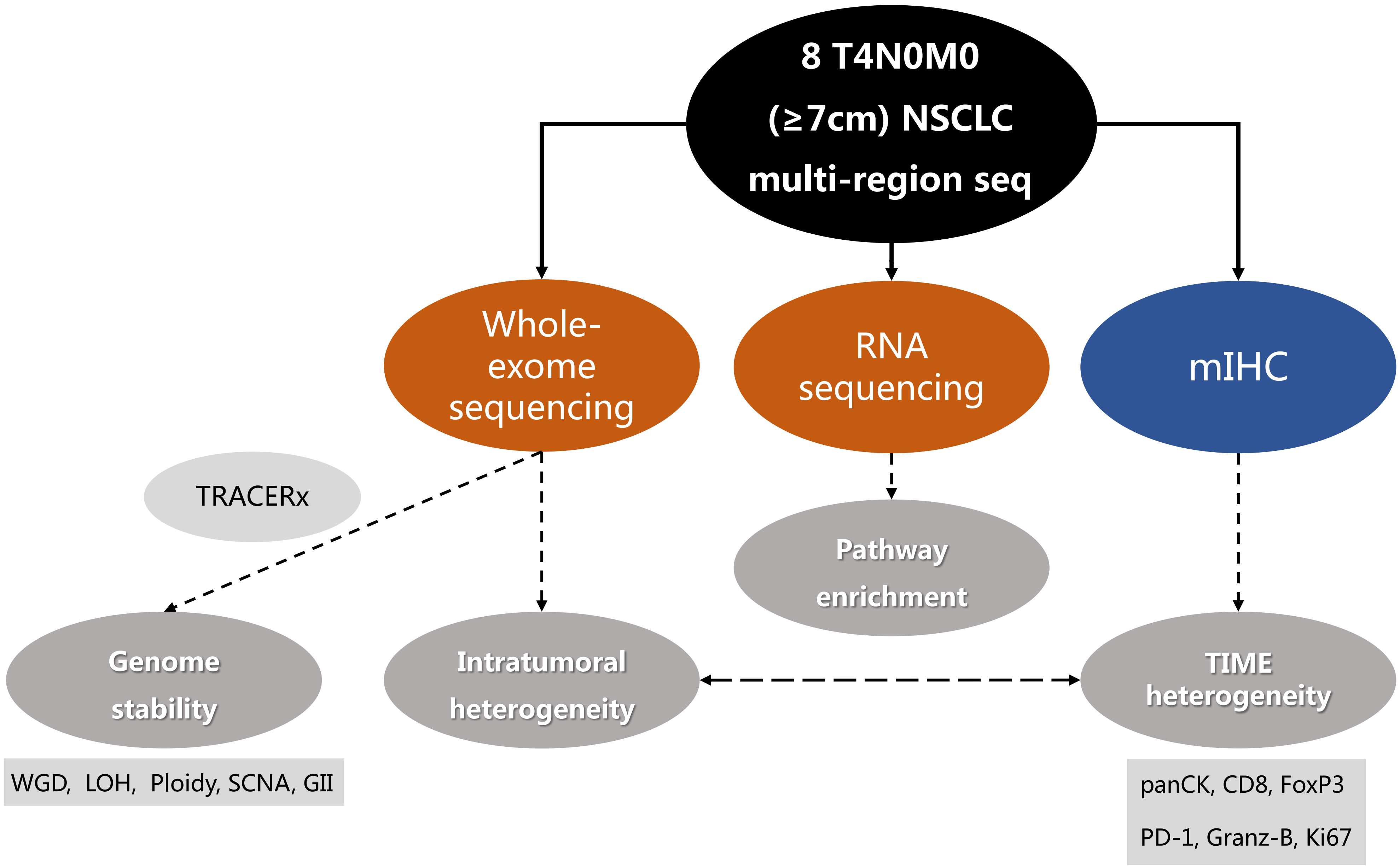

Supplementary Figure 2

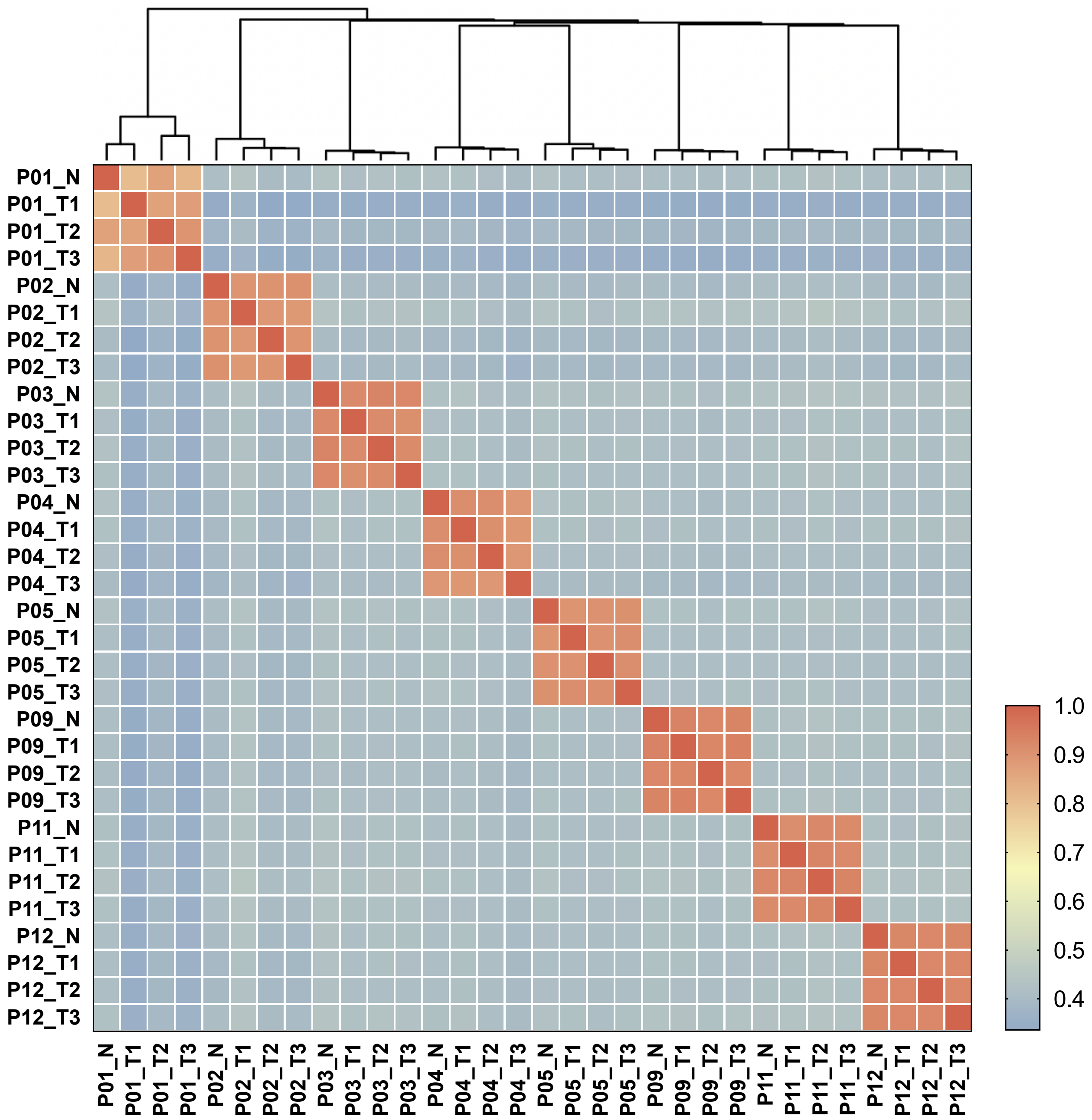

Supplementary Figure 3

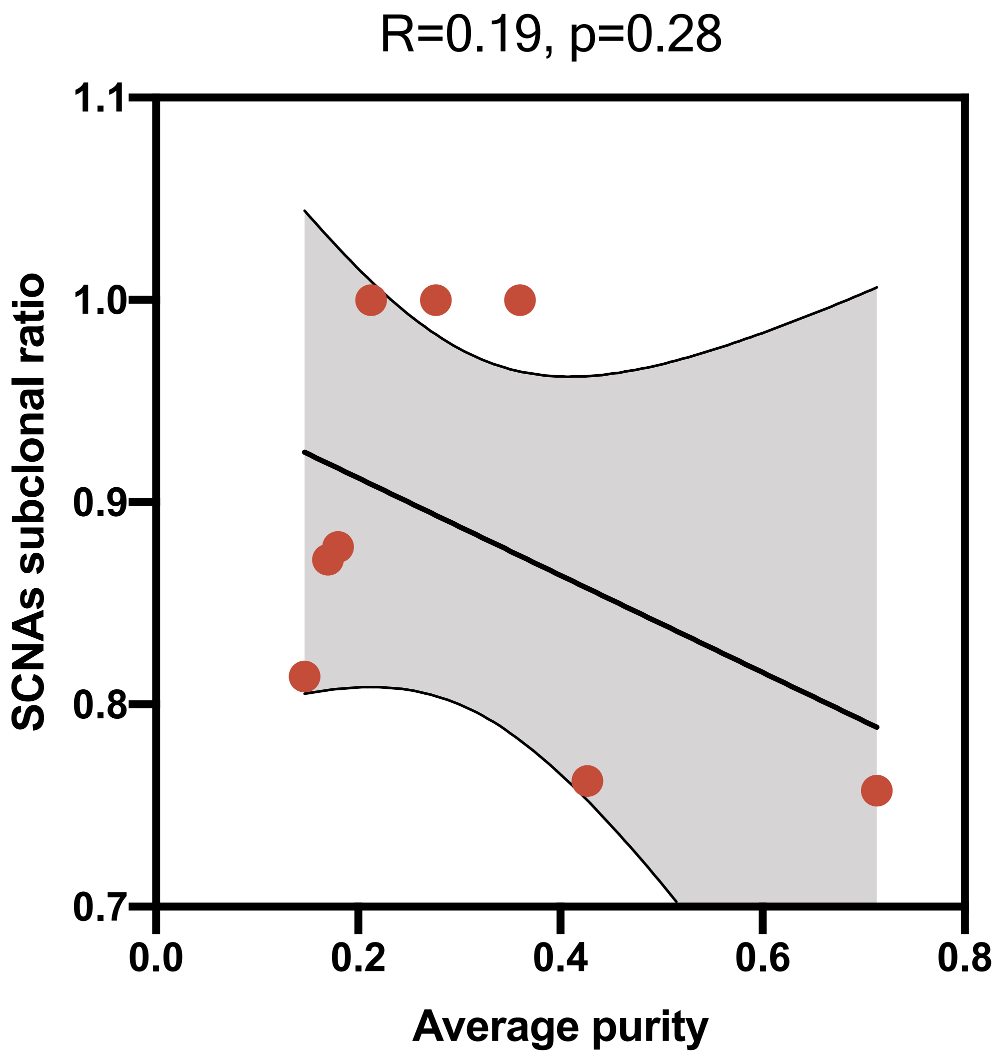

Supplementary Figure 4

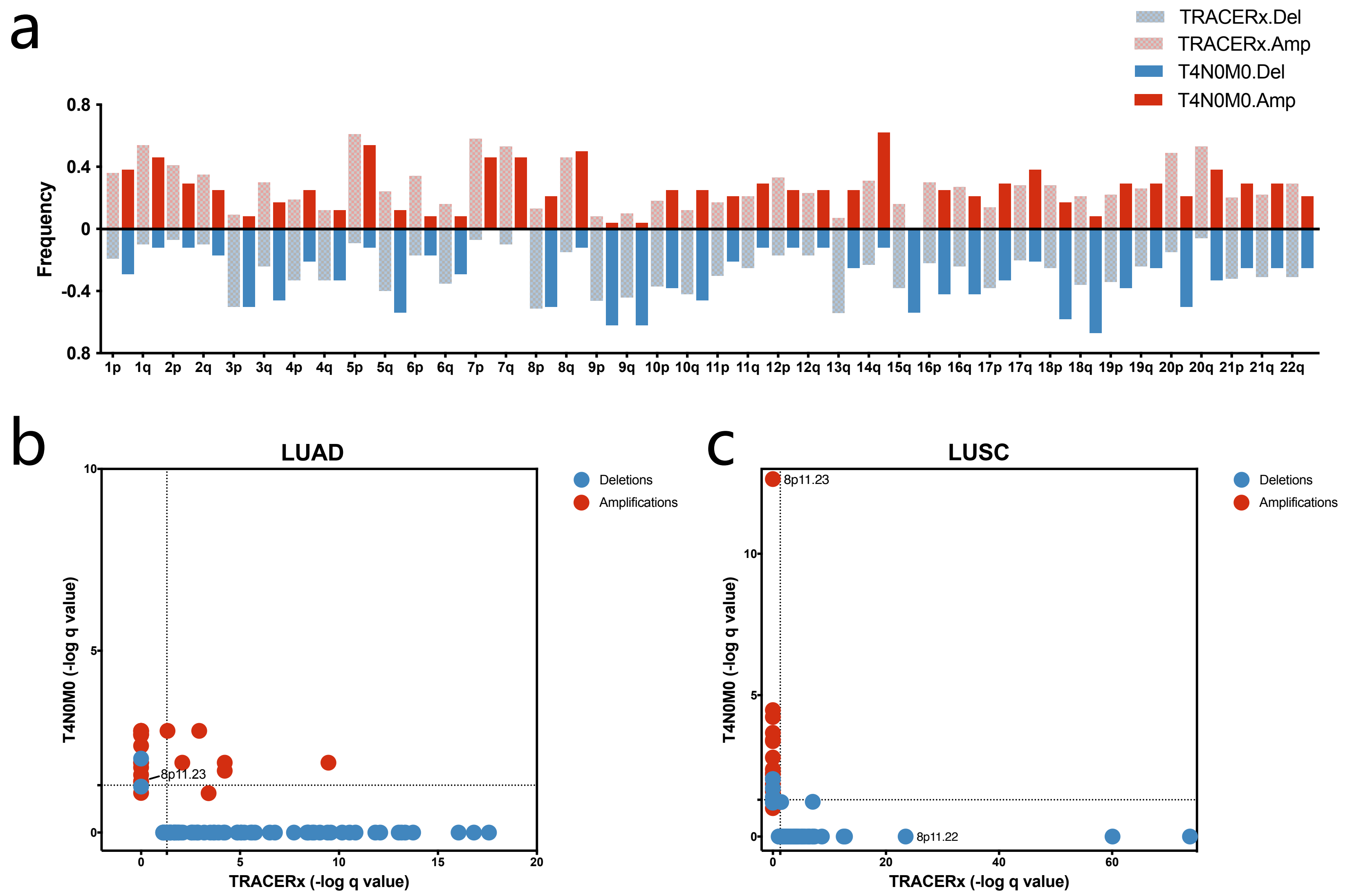

Supplementary Figure 5

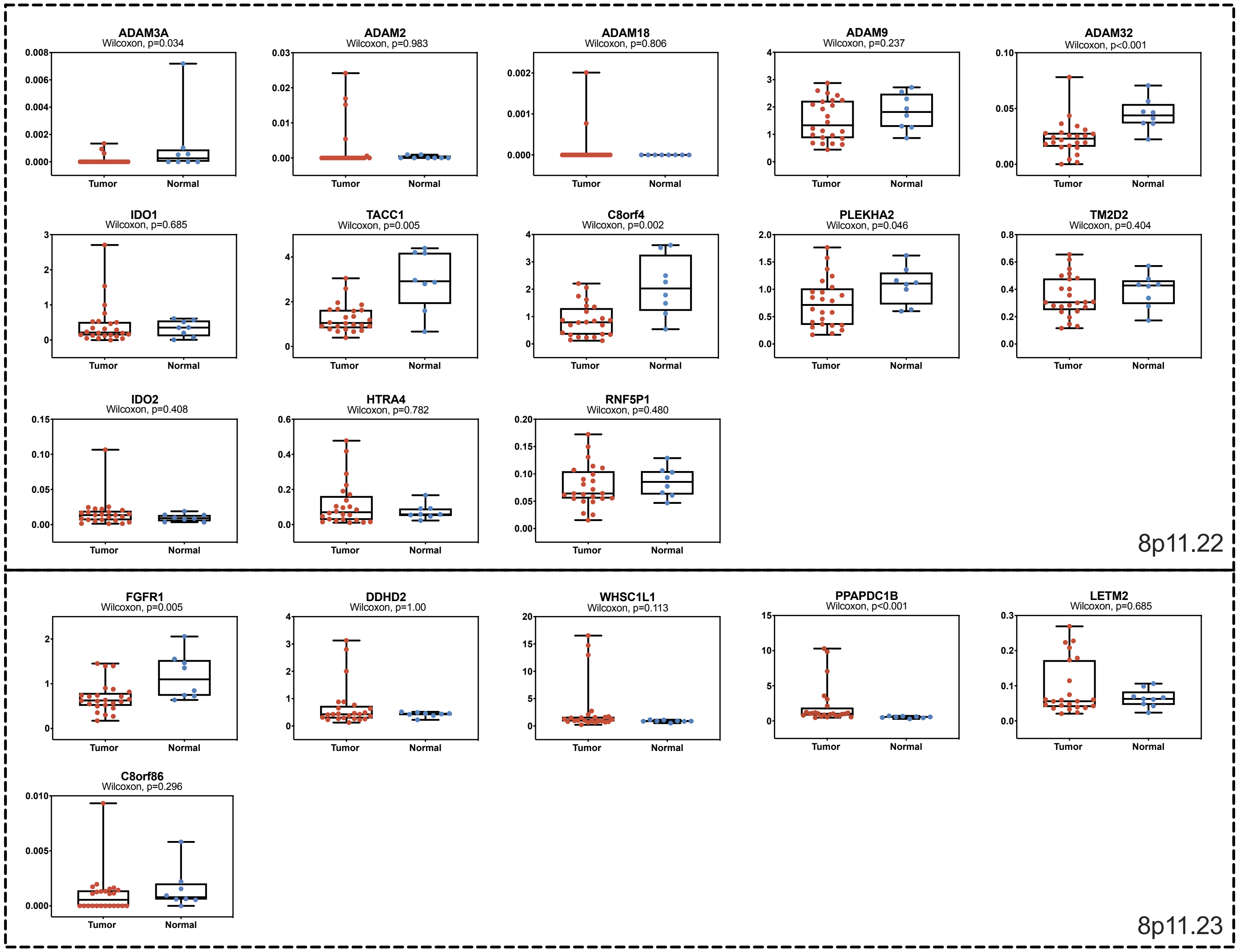

Supplementary Figure 6

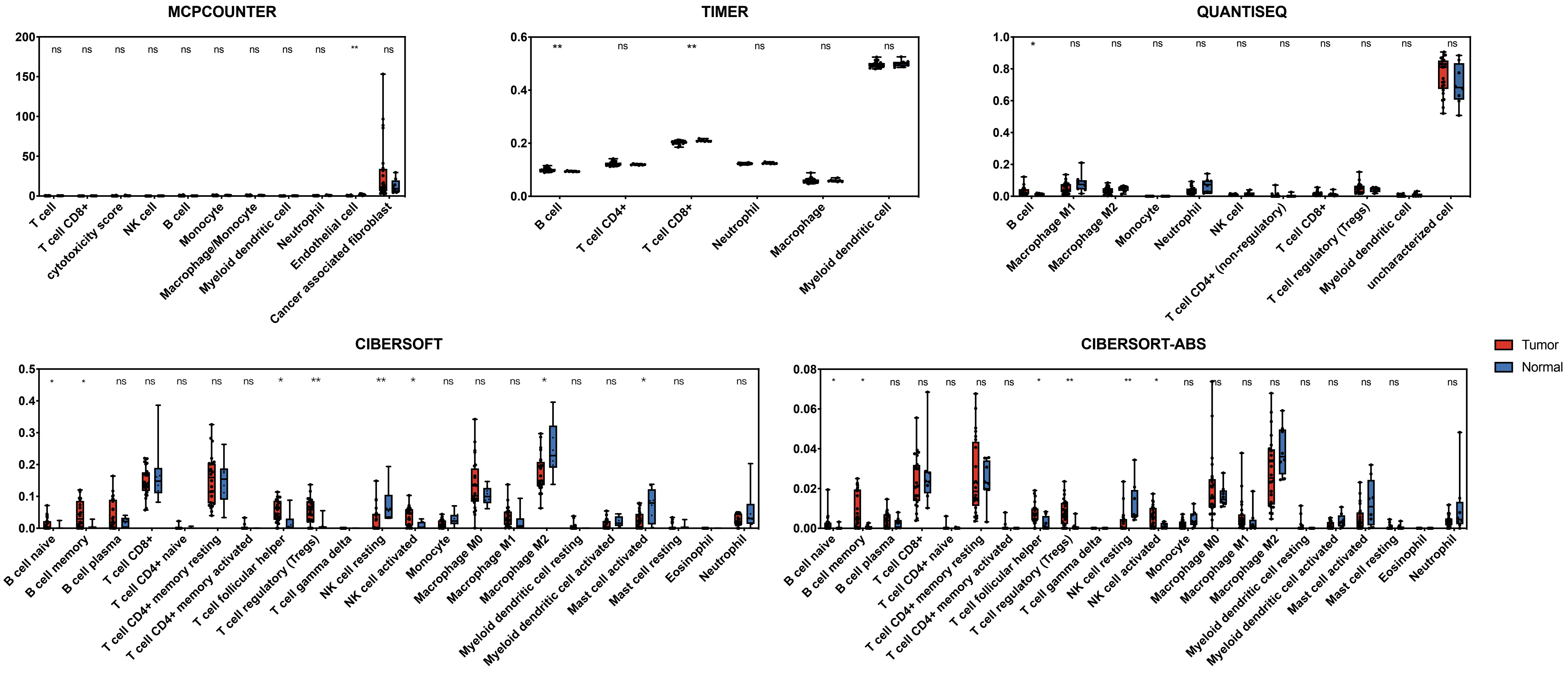

Supplementary Figure 7

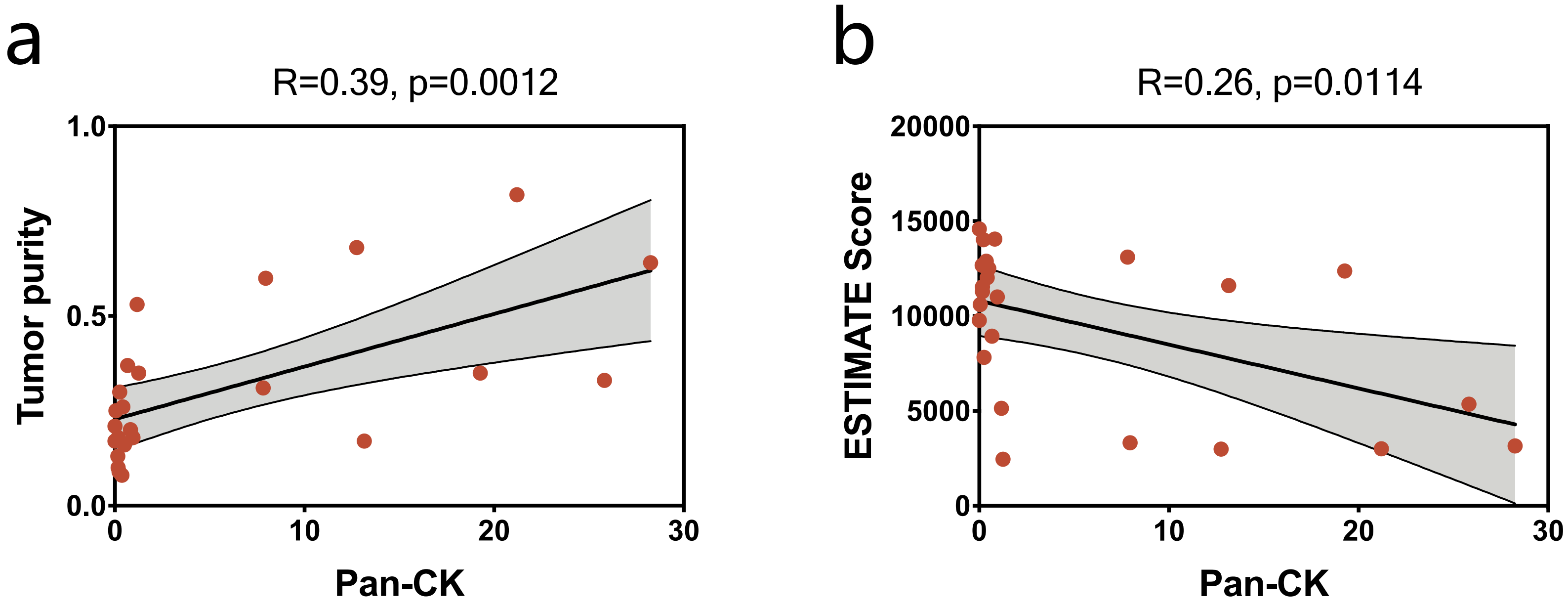

Supplementary Figure 8

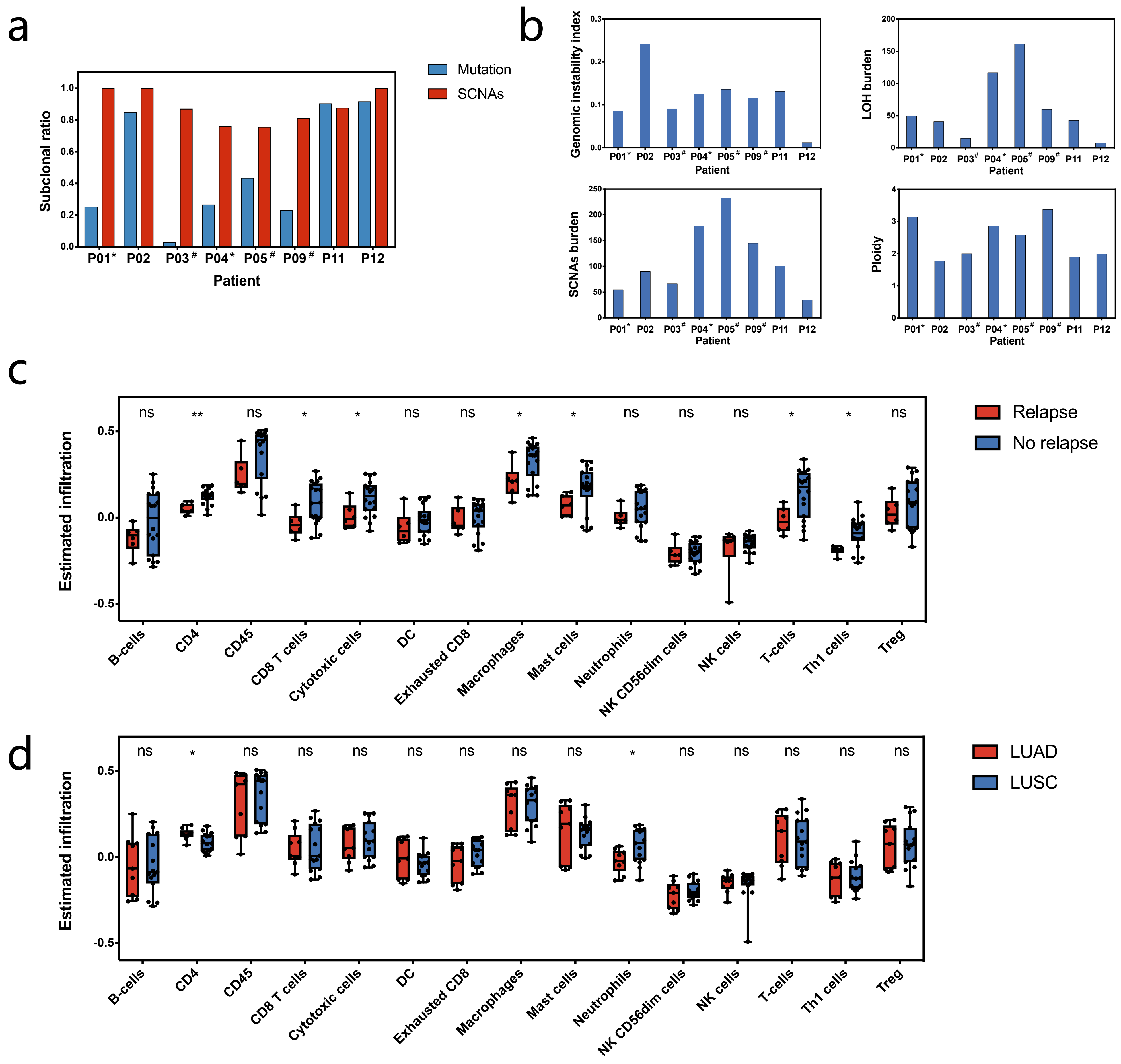

Supplement: Supplementary file 1 — Figure S1. Sequencing and analytical flowchart of the study. mIHC, multiplex immunohistochemistry; WGD, whole‐genome doubling; LOH, loss of heterozygosity; SCNA, somatic copy number alterations; GII, genomic instability index; TIME, tumor immune microenvironment. Figure S2. Cluster map of genetic distances between all samples. Figure S3. Correlation between somatic copy number alterations (SCNAs) and average tumor purity. Figure S4. (A) GISTIC amplification (top, red) and deletion (bottom, cyan) plots of the T4N0M0 tumor cohort (dark) and TRACERx cohort (light). (B) Amplification (red) and deletion (blue) q values from GISTIC2.0 for SCNA peaks of significant copy number gain and loss plotted for T4N0M0 adenocarcinomas versus TRACERx adenocarcinomas (LUAD, n = 61). (C) Amplification (red) and deletion (blue) q values from GISTIC2.0 for SCNA peaks of significant copy number gain and loss plotted for T4N0M0 squamous cell carcinomas (LUSC) versus TRACERx LUSCs. Figure S5. Gene expression levels of tumors (red) and adjacent normal tissues (blue) encompassing 8p11.22 and 8p11.23. Figure S6. Estimated proportion of infiltrating immune cells in tumors (red) and adjacent normal tissues (blue) obtained with MCPcounter, TIMER, QUANTISEQ, CIBORSORT, and CIBORSORT‐ABS. Figure S7. (A) Positive results of different mIHC markers between tumor regions and non‐tumor regions based on Pan‐CK signal. (B) Correlation analysis of tumor purity or ESTIMATE ratio with a Pan‐CK positive result. Figure S8. Subgroup analysis. (A) Subclonal mutation and somatic copy number alterations (SCNAs) ratio. (B) Genomic instability index, loss of heterozygosity (LOH), SCNAs burden and ploidy. (C) Abundance of different immune cell types estimated by ssGSEA methods, Relapse (n = 2) versus nonrelapse (n = 6). (D) Abundance of different immune cell types estimated by ssGSEA methods, lung adenocarcinoma (LUAD, n = 3) versus squamous cell carcinoma (LUSC, n = 5). The asterisk indicates relapsed patients and t [file TCA-13-1333-s002.pdf]
