# Supplementary material for: Intratumoral genetic and immune microenvironmental heterogeneity in T4N0M0 (diameter ≥ 7 cm) non‐small cell lung cancers
Source: Thorac Cancer. 2022 Apr 8;13(9):1333–41. doi: 10.1111/1759-7714.14393 (PMC9058296; doi:10.1111/1759-7714.14393)
Supplement: Supplementary file 2 — Table S1. Clinicopathological features of all eight enrolled patients. Table S2. Multiplex immunohistochemistry markers. [file TCA-13-1333-s001.docx]

| **Supplementary Table S1. Clinicopathologic features of all eight enrolled patients.** | | | | | | | | | | |
| --- | --- | --- | --- | --- | --- | --- | --- | --- | --- | --- |
|  | **Gender** | **Age, years** | **Smoking history^#^** | **Date of surgery** | **Histology type** | **Tumor site** | **Tumor size (cm)** | **Adjuvant therapy** | **Recurrence/ date** | **Recurrent site** |
| P01 | Male | 59 | 2 | Sep 13^th^, 2018 | SCC | RUL | 7.6 | Yes, four cycles of DP | Yes, Nov 10^th^, 2019 | Right lung multiple nodules, pleural effusion |
| P02 | Male | 57 | 1 | Nov 22^th^, 2018 | SCC | LLL | 9.0 | Yes, three cycles of GP | No | - |
| P03 | Male | 60 | 0 | Dec 6^th^, 2018 | AD | RUL | 7.4 | Yes, three cycles of DP | No | - |
| P04 | Male | 63 | 2 | Feb 19^th^, 2019 | SCC | LUL | 7.8 | No (pneumonectomy) | Yes, Oct 17^th^, 2019 | Multiple enlarged mediastinal lymph nodes |
| P05 | Male | 74 | 2 | Mar 28^th^, 2019 | AD | LLL | 7.2 | No (old age) | No | - |
| P09 | Male | 61 | 0 | May 7^th^, 2020 | AD | RUL | 8.1 | No (pneumonectomy) | No | - |
| P11 | Male | 57 | 1 | June 2^nd^, 2020 | SCC | RUL | 7.6 | Yes, only one cycle of DP | No | - |
| P12 | Male | 61 | 0 | Apr 23^th^, 2020 | SCC | RLL | 7.2 | No (patient refusal) | No | - |

^#^Smoking history: 0 for never smoker, 1 for previous smoker, 2 for current smoker; SCC: squamous cell carcinoma; AD: adenocarcinoma; RUL: right upper lobe; LLL: left lower lobe; LUL: left upper lobe; RLL: right lower lobe; DP: docetaxel plus carboplatin; GP: gemcitabine plus carboplatin; The follow‐up deadline was Dec 31^th^, 2021.

**Supplementary Table S2. Multiplex immunohistochemistry markers.**

| **Position** | **Antibody** | **Clone (host)** | **Dilution** | **Incubation** | **TSA dyes** | **Fluorescence stain** |
| --- | --- | --- | --- | --- | --- | --- |
| 1 | CD8 | CST #70306 | 1:200 | 1h | 480 | cyan |
| 2 | FoxP3 | CST #12653 | 1:400 | 1h | 520 | green |
| 3 | PD-1 | CST #86163 | 1:200 | 1h | 570 | yellow |
| 4 | Granz-B | CST #46890 | 1:125 | 1h | 620 | orange |
| 5 | Pan-CK | CST #4545T | 1:250 | 1h | 690 | red |
| 6 | Ki-67 | CST #9027 | 1:400 | 1h | 780 | purple |
| 7 | DAPI |  |  | 1h | DAPI | blue |

Formalin-fixed, paraffin-embedded tissues were cut into 4 µm-thick sections and stained using Opal Polaris 7 Color Automation IHC Detection Kit (Akoya Biosciences, Menlo Park, CA, USA) for the simultaneous detection and quantification of Pan-CK, CD8, FoxP3, PD-1, Granz-B, Ki-67, and DAPI (Supplementary Table 2). All 32 slides were observed and imaged using the Vectra Polaris automated quantitative pathology imaging system. The images were sequentially spectrally unmixed using Akoya Phenoptics inForm software (inform 2.4.8). Cellular quantification was performed using inForm software. Fluorescent DAPI signals were used to detect and separate each cell. The normalized fluorescence intensity in each channel was used to set a positive threshold for each biomarker. Regions of interest (ROIs) in the tumor and stroma were characterized based on Pan-CK signaling using a machine-learning tissue segmentation mechanism. The percentage of positive cells expressing individual markers was defined in the whole field of view as well as in the tumor and stroma ROIs.
